# A case report of pulmonary embolism following endoscopic cervical discectomy in an elderly patient with cervical disc herniation

**DOI:** 10.1097/MD.0000000000051120

**Published:** 2026-10-23

**Authors:** Shangchen Yu, Zheng Li, Shuguang Yang, Chao Chen, Kun Chen, Man Shi, Haiting Wang, Siqi Zhou, Wen Zhang, Xuebi Tian

**Affiliations:** a Department of Anesthesiology and Pain Medicine, Hubei Key Laboratory of Geriatric Anesthesia and Perioperative Brain Health, and Wuhan Clinical Research Center for Geriatric Anesthesia, Tongji Hospital, Tongji Medical College, Huazhong University of Science and Technology, Wuhan, Hubei, China.

**Keywords:** acute pulmonary embolism, case report, cervical disc herniation, endoscopic cervical discectomy

## Abstract

**Rationale:**

Posterior percutaneous endoscopic cervical discectomy (P-PECD) is a minimally invasive procedure for cervical spine disorders. Although it is associated with less surgical trauma and faster postoperative recovery, serious perioperative thromboembolic complications may still occur.

**Patient concerns:**

A 65-year-old woman with cervical disc herniation, right upper limb pain, and multiple comorbidities underwent posterior percutaneous endoscopic cervical discectomy after unsuccessful conservative treatment. On the 2nd postoperative day, she developed dizziness and decreased oxygen saturation. On the 3rd postoperative day, she complained of chest tightness.

**Diagnoses:**

Laboratory testing revealed a markedly elevated plasma D-dimer level. Venous color Doppler ultrasound of both lower limbs demonstrated thrombosis in the bilateral calf muscular veins, and computed tomography pulmonary angiography confirmed multiple pulmonary emboli.

**Interventions:**

The patient received oxygen therapy, continuous cardiorespiratory monitoring, anticoagulation with low-molecular-weight heparin, and other supportive treatments. She was transferred to the intensive care unit for further monitoring and management.

**Outcomes:**

The patient’s respiratory and circulatory status gradually stabilized, and her chest tightness and dizziness resolved. She was subsequently transferred back to the pain ward and discharged in stable condition.

**Lessons:**

This case highlights that minimally invasive spinal surgery does not eliminate the risk of perioperative venous thromboembolism. Comprehensive preoperative thrombotic risk assessment, individualized perioperative management of antithrombotic therapy, appropriate thromboprophylaxis, early mobilization, and timely recognition and treatment of suspected APE are important for improving perioperative safety.

## 1. Introduction

Cervical disc herniation (CDH) refers to a degenerative condition of the cervical intervertebral disc resulting from acute injury or chronic strain. It is characterized by compression of the spinal cord and/or nerve roots by the nucleus pulposus, resulting in clinical symptoms such as motor dysfunction and sensory disturbances. CDH may also manifest as scapular pain, which often precedes arm pain by several weeks.^[1]^ CDH is one of the common degenerative disorders of the cervical spine. This condition not only compromises the biomechanical integrity of the cervical spine but may also cause neurological dysfunction, thereby significantly reducing patients’ quality of life.

In recent years, endoscopic technology has advanced rapidly and is now widely used in the treatment of spinal diseases. Among these techniques, posterior percutaneous endoscopic cervical discectomy (P-PECD) allows precise targeting of the lesion, minimizes damage to cervical tissues, avoids injury to anterior structures such as the trachea, esophagus, carotid artery, jugular vein, thyroid, and recurrent laryngeal nerve. This approach offers benefits, including minimal surgical trauma, reduced intraoperative bleeding, shorter hospital stays, and fewer complications.^[2]^ P-PECD has been established as a safe and effective minimally invasive treatment for cervical spondylotic radiculopathy.^[3,4]^

Despite being minimally invasive, perioperative complications of P-PECD should not be overlooked. Among these, acute pulmonary embolism (APE), although rarely discussed, can be life-threatening if it occurs. APE typically results from obstruction of the pulmonary artery or its branches by a thrombus originating from the venous system or right heart, leading to impaired pulmonary circulation and right ventricular dysfunction. It ranks as the third most common cardiovascular disease globally and represents the most severe complication of venous thromboembolism (VTE), being the third leading cause of cardiovascular death.^[5–7]^

To the best of our knowledge, APE following P-PECD has not been previously reported in the published literature. This article presents a case of APE in an elderly patient after P-PECD at the Department of Pain, Tongji Hospital, Tongji Medical College, Huazhong University of Science and Technology. It provides a systematic analysis of the potential causes, diagnostic and therapeutic approaches, and prevention strategies for pulmonary embolism in this context. The goal is to raise awareness of APE risk following minimally invasive spinal surgery, enhance patient safety, and offer insights for optimizing perioperative management.

## 2. Clinical information

The patient was a 65-year-old woman, measuring 164 cm in height and weighing 71 kg. Around January 2024, she began experiencing pain and soreness in the right neck and shoulder without an obvious cause. The symptoms worsened with activity and improved with rest. She reported no fever, dizziness, headache, fatigue, or other associated symptoms and did not seek specific treatment at that time. On April 4, 2025, she developed soreness and weakness in the right upper limb without clear provocation. During the course of the disease, she experienced poor sleep and decreased appetite, although urination and bowel movements were normal, with no significant weight loss. Her medical history included hypertension for >5 years, which had been managed with oral nifedipine sustained-release tablets 20 mg twice daily, irbesartan tablets 150 mg once daily, and nicorandil tablets 5 mg 3 times daily. She also had a history of hyperlipidemia, for which she had been taking atorvastatin calcium tablets 10 mg once daily. Following coronary stent implantation, she had been receiving long-term antiplatelet therapy with a fixed-dose aspirin-containing combination product marketed in China as dihydroxyaluminum aminoacetate, heavy magnesium carbonate and aspirin tablets (II). Each tablet contained aspirin 81 mg, heavy magnesium carbonate 22 mg, and dihydroxyaluminum aminoacetate 11 mg, and was administered once daily. Additionally, she had coronary atherosclerotic heart disease and had been receiving rosuvastatin/ezetimibe tablets (10/10 mg) once daily.

Physical examination showed that the patient was conscious and cooperative. Bilateral pupils were equal in size (approximately 4 mm in diameter) with a reactive light reflex. Eye movement was normal, the forehead and nasolabial folds were symmetrical, and the tongue was midline. Cervical spine flexion and extension were limited, and the head percussion test was positive. Breath sounds were slightly coarse bilaterally, with no dry or wet rales detected. Heart rhythm was regular, heart sounds were normal, and no pathological murmurs were heard. The abdomen was soft, without tenderness, rebound pain, or palpable masses; bowel sounds were normal. No spinal deformity was observed. Limb muscle strength and tone were normal, sensory function was intact, no pathological signs were elicited, and other examinations were unremarkable. The numerical rating scale score at admission was 8/10 (Fig. 1).

**Figure 1.**
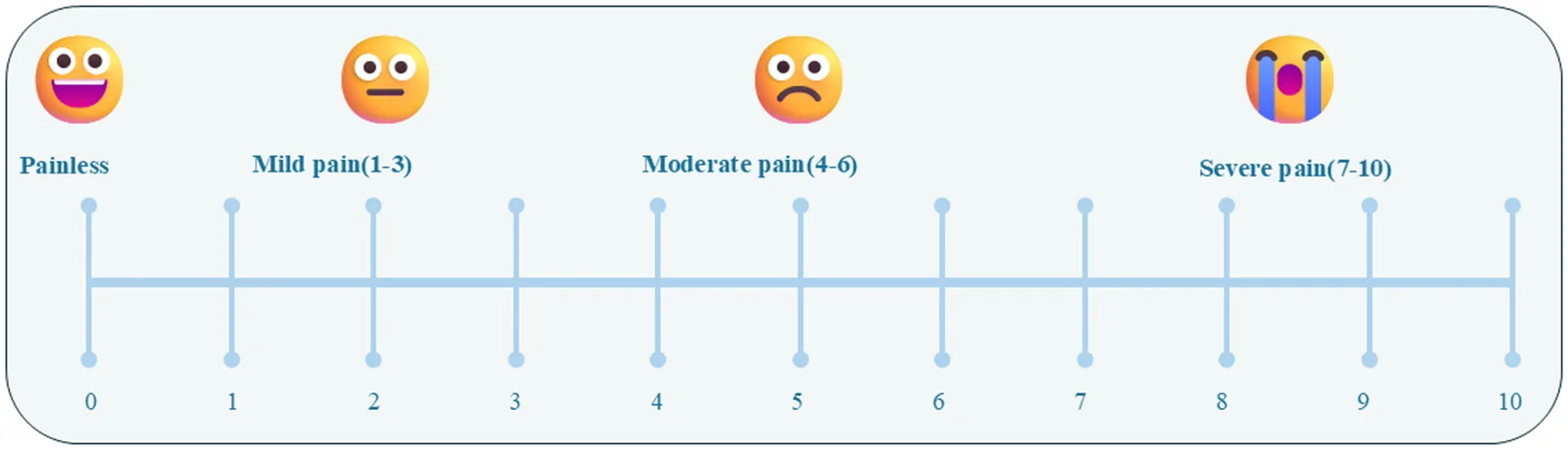
The numerical rating scale. The numerical rating scale consists of 11 ratings. According to the selection results, it can be divided into no pain (0 points), mild pain (1–3 points), moderate pain (4–6 points), and severe pain (7–10 points).

Preoperative laboratory investigations (April 14) revealed normal results for complete blood count, liver function, renal function, electrolytes (K^+^, Na^+^, Cl^−^, Ca^2+^), blood glucose, lipid profile, coagulation panel, high-sensitivity cardiac troponin I, myoglobin, creatine kinase MB isoenzyme, high-sensitivity C-reactive protein, and pretransfusion 8-item screen. Plasma D-dimer was elevated at 0.80 μg/mL fibrinogen equivalent units (FEU). Cervical spine magnetic resonance imaging plain scan (April 10) demonstrated degenerative changes, with a prominent posterior protrusion of the C3/4 intervertebral disc causing compression of the thecal sac and spinal cord at that level. Mild disc protrusions were also noted at C4/5 and C5/6. Magnetic resonance imaging of the right shoulder (April 11) indicated bone marrow edema at the tendon attachment site of the humeral head, acromioclavicular arthritis, an injury to the supraspinatus tendon with a partial tear, as well as lesions involving the intra-articular portion of the long head of the biceps tendon and the subscapular tendon. A small amount of effusion was observed in the right glenohumeral joint cavity, the subcoracoid bursa, and the suprascapular recess. A 12-lead electrocardiogram was within normal limits. Anteroposterior chest radiography revealed an enlarged cardiac silhouette. Color Doppler ultrasound of the arterial and venous systems of both lower limbs showed multiple atherosclerotic plaques in the arteries.

Upon admission, the patient was administered intravenous dexamethasone sodium phosphate (5 mg), intravenous mannitol (250 mL), and intravenous mecobalamin (0.5 mg) for acute pain management. Oral ebastine tablets (10 mg once daily) and topical halometasone cream (twice daily) were prescribed for anti-allergy therapy. Dihydroxyaluminum aminoacetate, heavy magnesium carbonate, and aspirin tablets (II) were discontinued and replaced with subcutaneous enoxaparin sodium injection (4000 U twice daily) and oral ibuprofen and codeine phosphate tablets (1 tablet twice daily). After ruling out surgical contraindications, a diagnostic block was performed under local anesthesia on April 18 using 1% lidocaine (1 mL injected into the right C5/6 intervertebral foramen). The patient reported over 50% symptomatic relief in the right upper extremity following the surgery. After assessment of anesthetic and surgical risks, the patient underwent endoscopic right C5/6 cervical discectomy, spinal nerve root adhesion release, discoscopic laminoplasty, and partial resection of the ligamentum flavum under general anesthesia on April 21. Intraoperative vital signs remained stable, and the procedure was uneventful. The patient recovered normally from anesthesia. Postoperatively, the patient was fitted with a neck brace and instructed to maintain strict bed rest in the supine position for 24 hours, during which passive lower limb exercises were performed. After this period, the patient was permitted to ambulate gradually while wearing the cervical collar.

On the 1st postoperative day (April 22), the patient’s right upper limb symptoms were significantly relieved. The patient reported no panic, chest tightness, or other discomforts. Oral medications were administered, including dihydroxyaluminum aminoacetate, heavy magnesium carbonate, and aspirin tablets (II) once daily and ibuprofen and codeine phosphate tablets (1 tablet twice daily) for analgesia, mecobalamin tablets 0.5 mg (1 tablet 3 times daily) for neurotrophic support, and horse chestnut seed extract tablets (1 tablet twice daily) to reduce edema.

On the 2nd postoperative day (April 23), the patient experienced dizziness after several trips to the toilet, accompanied by chest tightness when sitting or standing; symptoms were relieved by lying down. Cardiac monitoring was initiated. The patient’s saturation of pulse oximetry (SpO_2_) gradually decreased to 94% without oxygen support and dropped further to 85% after activity. With mask oxygen inhalation at 7 L/min, SpO_2_ reached 97% (compared to 97% and 96% preoperatively). Heart rate and blood glucose were measured immediately and were within the normal ranges. Blood pressure measured immediately after the onset of dizziness was 106/72 mm Hg (admission blood pressure: 124/75 mm Hg). After 5 minutes, it decreased to 92/54 mm Hg, which was lower than previous readings, and later returned to 115/67 mm Hg.

On the 3rd postoperative day (April 24), the patient’s condition remained largely unchanged. Chest digital radiography revealed an enlarged cardiac shadow. Laboratory tests showed elevated myoglobin, creatine kinase MB isoenzyme, high-sensitivity cardiac troponin I (111.4 pg/mL), and N-terminal pro-B-type natriuretic peptide (2111.0 pg/mL). Subcutaneous enoxaparin sodium injection (4000 U) was administered for anticoagulant therapy. Arterial blood gas analysis indicated a pH of 7.474, partial pressure of carbon dioxide (PaCO_2_) of 30.3 mm Hg, partial pressure of oxygen (PaO_2_) of 164 mm Hg, actual bicarbonate of 22.0 mmol/L, and SpO_2_ of 99.2%. The plasma D-dimer level was significantly elevated at 15.39 μg/mL FEU. Echocardiography showed no obvious abnormalities. However, venous color Doppler ultrasound of both lower limbs demonstrated venous thrombosis within the bilateral calf muscle groups. The patient was transferred to the Department of Critical Care Medicine. The thrombelastogram showed no significant abnormalities. Anticoagulant therapy with subcutaneous enoxaparin sodium (4000 U q12h) was continued, and right femoral vein catheterization was performed. Computed tomography pulmonary angiogram (CTPA) confirmed multiple pulmonary emboli in bilateral 2nd- to 3rd-order pulmonary artery branches. Additional findings included minor exudative changes and partial atelectasis in both lower lobes, bilateral pleural thickening and adhesions, aortic wall calcification, and striated hyperdense shadows along the coronary artery tracts (Fig. 2).

**Figure 2.**
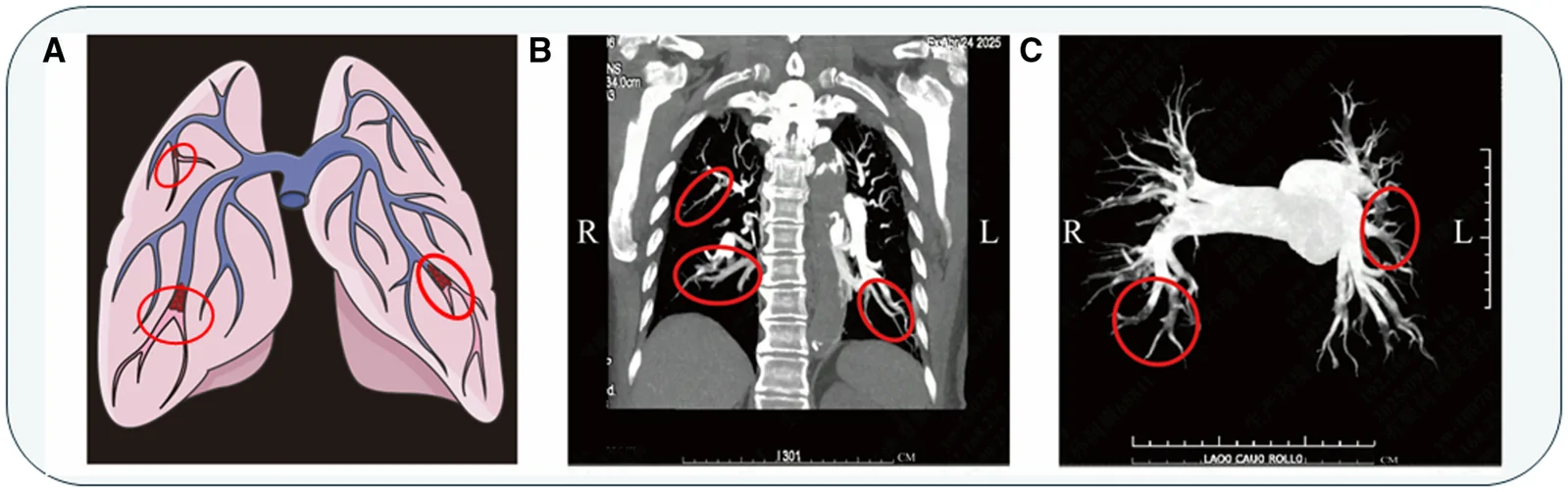
Computed tomography pulmonary angiography and schematic illustration of pulmonary emboli. (A) Schematic illustration showing the locations of pulmonary emboli. (B) Coronal computed tomography pulmonary angiography image. (C) Three-dimensional reconstruction of the pulmonary arteries. Red circles indicate the locations of pulmonary emboli.

On April 25, the patient was conscious and in fair general condition, receiving high-flow nasal oxygen at 40 L/min. Vital signs included a heart rate of 64 beats/min, respiratory rate of 18 breaths/min, blood pressure of 126/68 mm Hg, and oxygen saturation of 100%. Both pupils were equal and round, approximately 4.0 mm in diameter, with intact light reflexes. Breath sounds were slightly coarse bilaterally, with no significant dry or wet rales detected. Heart sounds were clear and rhythm regular, without obvious pathological murmurs. The abdomen was soft, non-tender, and without rebound pain; bowel sounds were present, and the shifting dullness test was negative. There was no edema in the lower limbs. Muscle strength and tone were normal, and no pathological signs were observed. Laboratory tests revealed a white blood cell count of 11.41 × 10^9^/L, with an absolute neutrophil count of 9.22 × 10^9^/L, absolute lymphocyte count of 1.22 × 10^9^/L, and absolute monocyte count of 0.84 × 10^9^/L. Red blood cell count was 3.84 × 10^12^/L, hemoglobin 116 g/L, and platelet count 175 × 10^9^/L. Liver and kidney function were within normal limits. Platelet autoantibody and alloantibody tests were negative. Plasma D-dimer was elevated at 17.36 μg/mL FEU. The patient continued to receive anticoagulation, gastric protection, oxygen support, and symptomatic treatment to prevent complications. Endotracheal intubation and mechanical ventilation were prepared if required. A cardiology consultation noted that the patient maintained acceptable oxygen saturation with high-flow nasal oxygen, with no significant hemodynamic instability. Anticoagulation with enoxaparin sodium injection (4000 U subcutaneously q12h) was continued, and follow-up pulmonary CTA was recommended in 3 to 5 days.

On April 26, the patient’s SpO_2_ was 91% while receiving high-flow nasal oxygen at 30 L/min. The remainder of the clinical condition was unchanged. Anticoagulation therapy was maintained. Laboratory results were as follows: white blood cell count 8.54 × 10^9^/L, absolute neutrophil 5.71 × 10^9^/L, absolute lymphocyte 2.08 × 10^9^/L, absolute monocyte 0.43 × 10^9^/L, red blood cell count 3.63 × 10^12^/L, hemoglobin 108.0 g/L, and platelet count 165.0 × 10^9^/L.

On April 27, the patient remained conscious and mentally alert, with SpO_2_ maintained at 98% under nasal cannula oxygen at 5 L/min. Respiratory and circulatory status were stable, and physical examination findings were consistent with previous assessments. Repeat complete blood count showed: white blood cell count 7.93 × 10^9^/L, absolute neutrophils 5.17 × 10^9^/L, absolute lymphocytes 1.92 × 10^9^/L, absolute monocytes 0.47 × 10^9^/L, red blood cell count 3.73 × 10^12^/L, hemoglobin 110.0 g/L, and platelet count 200.0 × 10^9^/L. Coagulation studies, including prothrombin time, prothrombin activity, international normalized ratio, and activated partial thromboplastin time, were within normal ranges. Fibrinogen was 4.64 g/L, thrombin time was 24.5 seconds, and plasma D-dimer was 4.41 μg/mL FEU.

On April 28, the patient was conscious and receiving oxygen via nasal cannula (2 L/min), with SpO_2_ maintained at 100%. Respiratory and circulatory status remained stable. The patient was transferred back to the pain ward for continued management. Anticoagulation therapy with enoxaparin sodium injection (4000 U q12h) was continued. Additional medications included ibuprofen and codeine phosphate tablets (1 tablet twice daily) for analgesia, mecobalamin tablets (0.5 mg 3 times daily) for neurosupport, and horse chestnut seed extract tablets (2 tablets twice daily) for edema reduction. Pain in the right neck, shoulder, and upper extremity had improved compared to prior assessments.

On April 29, the patient’s condition remained stable without significant change. By April 30, the patient reported no episodes of panic, chest tightness, or other discomfort. Sleep quality was good, and the patient was in positive spirits. Swelling and pain in the right neck, shoulder, and upper extremity had significantly decreased. The patient was discharged on medical advice (Fig. 3).

**Figure 3.**
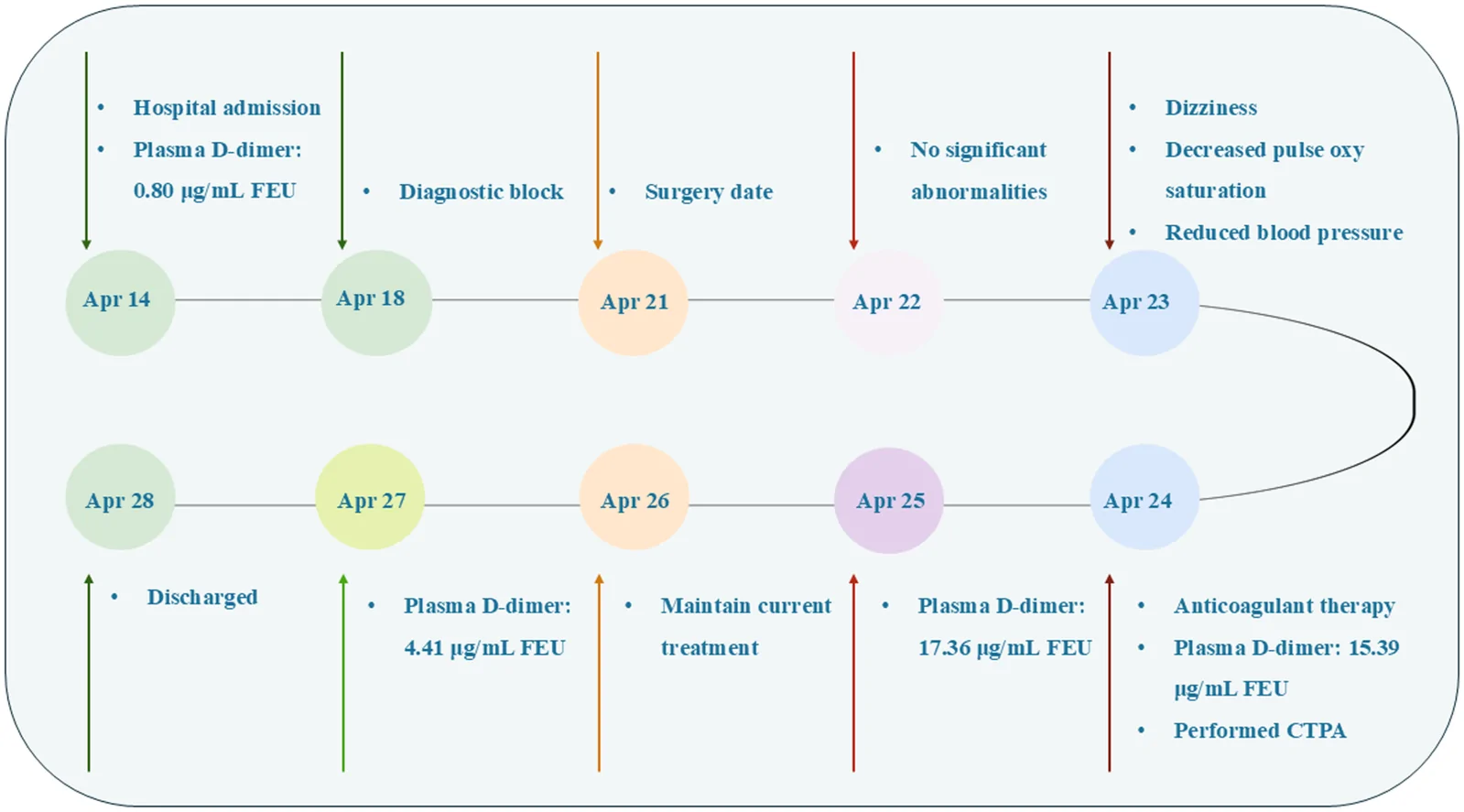
Clinical timeline and D-dimer dynamics during perioperative period. CTPA = computed tomography pulmonary angiography, FEU = fibrinogen equivalent units.

## 3. Analysis of possible causes of postoperative pulmonary embolism

This patient was indicated for P-PECD due to CDH that significantly impacted daily living and sleep. Following the procedure, the patient experienced notable pain relief. However, APE occurred as a postoperative complication. Fortunately, with timely interventions, including anticoagulation therapy, respiratory support, and other symptomatic and supportive measures, the patient did not develop hemodynamic instability or other serious complications. The patient had a favorable recovery without sequelae and was eventually discharged in stable condition.

### 3.1. Advanced age and multiple comorbidities

Underlying disease may play an important role in the development of APE. Hypertension, for instance, alters blood flow shear stress and increases oxidative stress, leading to endothelial dysfunction. This promotes vascular inflammation and remodeling, thereby elevating the risk of thrombosis.^[8,9]^ Long-term elevated blood pressure also predisposes hypertensive patients to atherosclerosis and plaque formation. These lesions not only impair blood circulation but may also facilitate localized thrombus formation. Similarly, hyperlipidemia contributes to elevated blood viscosity, and abnormal lipid metabolism further exacerbates this condition, increasing the likelihood of thrombosis. Notably, obese patients often exhibit a hypercoagulable state.^[10,11]^ In elderly patients, age-related decline in physiological function often disrupts the balance between the coagulation and fibrinolytic systems, resulting in a greater tendency toward hypercoagulability. In this case, the patient was a 65-year-old woman with a body mass index of 26.4 kg/m^2^. She had multiple comorbidities, including hypertension, hyperlipidemia, coronary atherosclerotic heart disease, and a history of coronary stent implantation. These conditions may have contributed to endothelial dysfunction and an increased thrombotic risk.

Additionally, her coronary artery disease required long-term antiplatelet therapy (e.g., dihydroxyaluminum aminoacetate, heavy magnesium carbonate, and aspirin tablets (II)). The perioperative adjustment of her antithrombotic regimen, including discontinuation of the antiplatelet agent and administration of low-molecular-weight heparin, may have introduced fluctuations in coagulation parameters. Together, these factors may have contributed to the development of postoperative acute pulmonary embolism in this patient.

### 3.2. Preoperative D-dimer abnormalities and the effects of fasting

Preoperative assessment of coagulation parameters is crucial for evaluating a patient’s thrombotic risk. As a degradation product of fibrin, elevated D-dimer levels are closely related to an increased risk of deep vein thrombosis (DVT) and other thrombotic events. Studies have indicated that elevated preoperative D-dimer is a significant predictor of acute or subacute postoperative DVT in cancer patients.^[12]^ In this case, the patient’s preoperative D-dimer level was 0.80 μg/mL FEU, which was slightly higher than the normal reference value of 0.5 μg/mL FEU. Additionally, color Doppler ultrasound of both lower limbs revealed multiple atherosclerotic plaques, indicating underlying vascular disease that may have contributed to the patient’s overall thrombotic risk. Unfortunately, these signs did not receive sufficient clinical attention before surgery.

Fasting and fluid restriction are standard measures before general anesthesia. However, the physiological changes induced by these protocols can adversely affect coagulation status. Reduced fluid intake leads to hemoconcentration, increased blood viscosity, and decreased blood flow velocity. These alterations enhance platelets’ contact with the vascular endothelium and further promote activation of the coagulation cascade.

### 3.3. Effects of surgical trauma and postoperative pain

Surgical trauma-induced vascular injury is a key factor in activating the coagulation system. Intraoperative manipulation inevitably causes damage to vascular endothelial cells, leading to exposure of subendothelial collagen fibers and basement membrane components. This, in turn, triggers platelet adhesion and activation of the coagulation cascade.^[13,14]^

Furthermore, acute postoperative pain not only induces significant patient stress but also promotes a hypercoagulable state through multiple pathways, thereby increasing thrombosis risk.^[13,15]^ Studies have demonstrated that a multimodal analgesia approach, such as postoperative electroacupuncture combined with continuous computed tomography-guided femoral nerve block, can effectively reduce the incidence of deep vein thrombosis following total knee arthroplasty.^[16]^ Such strategies not only provide sufficient analgesia but also mitigate pain-related stress and hypercoagulability. In the present case, postoperative pain management relied solely on oral analgesics. This approach may have been suboptimal given the generally lower compliance among elderly patients and potential inadequacy in pain control. Insufficient analgesia may, therefore, have contributed to an increased thrombotic risk in this patient.

### 3.4. Effects of postoperative mobility restrictions

Studies indicate that patients with limited preoperative mobility exhibit a significantly higher incidence of APE within 30 days after surgery.^[17]^ This correlation is closely associated with venous stasis resulting from postoperative immobility. In this case, the patient was instructed to maintain strict bed rest for 24 hours following the procedure. Although passive or active lower limb exercises were recommended during this period, adherence is often suboptimal among elderly patients. Furthermore, neck brace immobilization further restricted overall mobility. Collectively, these factors may have promoted venous stasis and increased the risk of thrombosis.

## 4. Prevention and treatment of acute pulmonary embolism

In this patient, early recognition and timely intervention were essential after the occurrence of APE.

### 4.1. Preoperative identification of risk factors and risk assessment

Risk factors for APE can be categorized based on odds ratio (OR) into strong (OR > 10), moderate (OR 2–9), and weak (OR < 2) predisposing factors.^[18,19]^ These include lower extremity fracture, major trauma, hip or knee arthroplasty, myocardial infarction within the past 3 months, history of VTE, spinal cord injury, chemotherapy, blood transfusion, congestive heart failure or respiratory failure, hormone replacement therapy, malignancy, prolonged bed rest (>3 days), diabetes, hypertension, and obesity. In this case, the patient was not only elderly and had a high body mass index but also presented multiple risk factors, such as hypertension, hyperlipidemia, coronary atherosclerotic heart disease, and a history of coronary stent implantation.

Preoperative evaluation should include coagulation profiles, D-dimer testing, and lower extremity arteriovenous Doppler ultrasound. Studies have shown that in patients undergoing primary total knee arthroplasty, a postoperative D-dimer level ≥1.0 mg/L is associated with a significantly increased risk of postoperative DVT, suggesting that D-dimer can serve as an important predictor of thrombotic risk.^[20]^ Particularly, D-dimer levels exceeding 1.5 μg/mL should raise clinical concern for possible underlying VTE.^[21]^

Preoperative assessment of the clinical probability of pulmonary embolism is essential for APE prevention. Comprehensive evaluation incorporating patient age, comorbidities, and coagulation status allows more accurate identification of high-risk individuals and facilitates targeted interventions. Two widely used clinical prediction tools are the Wells score (Table 1)^[22]^ and the modified Geneva score (Table 2).^[23]^ These scales incorporate symptoms, signs, and risk factors, including age, heart rate, medical and surgical history, and clinical features, to stratify patients into different risk categories. Emerging scoring systems continue to improve the accuracy of pulmonary embolism diagnosis. For example, the Shanxi Dayi Hospital score, developed by the First Clinical Hospital of Shanxi Medical University, has shown high specificity and superior calibration and discrimination compared to traditional scores in the Chinese population.^[24]^ Additionally, the “YEARS” algorithm, a validated diagnostic model for APE, has been shown to reduce the need for CTPA in nearly half of suspected APE cases when compared to the Wells score (Fig. 4).^[25,26]^ Furthermore, specific exclusion criteria can help determine whether pulmonary embolism screening is warranted.^[27]^

**Table 1:** Scoring of the 7 variables of the Wells rule variable.

Variable	Points
Clinical signs and symptoms of DVT (minimum of leg swelling and pain on deep venous palpation)	3
Alternative diagnosis is less likely than PE	3
Heart rate >100 beats/min	1.5
Immobilization or surgery in the previous 4 wk	1.5
Previous DVT/PE	1.5
Hemoptysis	1
Malignant neoplasm(patient receiving treatment, treated in the past 6 mo, or receiving palliative care)	1

The possibility of suffering from pulmonary embolism: a total score of 0 to 1 indicates low possibility, 2 to 6 indicates moderate possibility, and ≥7 indicates a high likelihood of suffering from VTE.

DVT = deep venous thrombosis, PE = pulmonary embolism.

**Table 2:** Scoring of the 8 variables in the original and simplified revised Geneva score.

Variable	Original	Simplified
Age >65 yr	1	1
Previous DVT or PE	3	1
Surgery (under general anesthesia) or fracture (of lower limbs) within 1 mo	2	1
Active malignant condition (solid or hematologic, currently active or considered cured <1 yr)	2	1
Unilateral lower-limb pain	3	1
Hemoptysis	2	1
Heart rate, beats/min		
75–94	3	1
≥95	2	1
Pain on lower-limb deep venous palpation and unilateral edema	4	1

For the original version of the scoring criteria, a total score of 0 to 3 points indicated a low possibility of pulmonary embolism, 4 to 10 points indicated moderate possibility, and ≥11 points indicated high possibility; for the simplified version of the scoring criteria, 0 to 1 points indicate low possibility, 2 to 4 points indicate moderate possibility, and ≥5 points indicate high possibility.

DVT = deep venous thrombosis, PE = pulmonary embolism.

**Figure 4.**
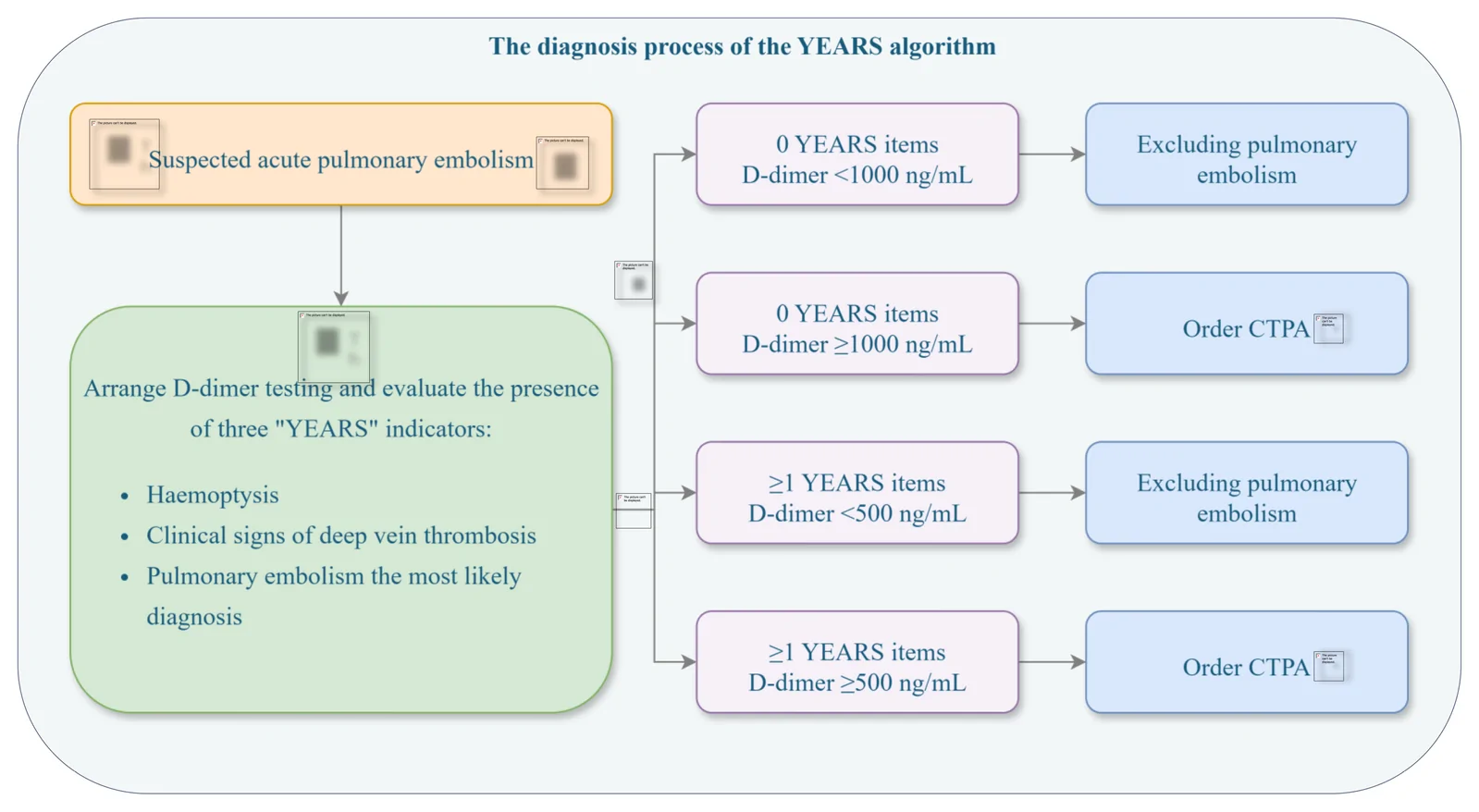
The diagnostic process of the YEARS algorithm. The algorithm reduces unnecessary CTPA examination by measuring D-dimer and combining the evaluation of 3 “YEARS” indicators. CTPA = computed tomography pulmonary angiogram.

### 4.2. Preoperative standardization of anticoagulation management

Endoscopic cervical discectomy is a minimally invasive procedure designed to reduce surgical trauma and tissue damage. While it offers advantages such as less muscle injury, reduced bleeding, shorter operative time, and faster recovery, the risk of postoperative hematoma formation remains a significant concern.^[28]^ This complication can lead to serious neurological deficits and may necessitate emergency reoperation.^[29]^ Additionally, the procedure carries a potential risk of vascular injury, especially to the vertebral artery.

The present case involves a patient with multiple underlying comorbidities and a history of coronary stent implantation, requiring long-term antiplatelet therapy. However, continuing anticoagulants during the perioperative period increases the risk of surgical bleeding. It is therefore essential for clinicians to thoroughly evaluate the bleeding risk associated with the surgery and to develop an individualized preoperative anticoagulation strategy. This includes the appropriate selection and timing of alternative anticoagulant therapies based on pharmacological profiles. For patients taking direct oral anticoagulants (DOACs), a standardized management approach is recommended in elective surgeries. DOACs may be continued for procedures with low bleeding risk. For those with moderate-to-low bleeding risk, discontinuation 1 day before surgery and resumption 1 day postoperatively is advised. In high-bleeding-risk surgeries, DOACs should be stopped 2 days preoperatively and resumed 2 days after surgery. Routine monitoring of DOAC levels or heparin bridging is generally not required. In emergency, urgent, or semi-urgent scenarios where DOAC levels are elevated or unknown, reversal agents should be considered.^[30]^

### 4.3. Intraoperative protocols, supportive measures, and close monitoring

Surgical trauma and perioperative physiological stress may contribute to postoperative hypercoagulability. Therefore, careful surgical manipulation, minimization of unnecessary tissue injury, and appropriate control of operative duration are important considerations for reducing thrombotic risk. Intraoperative thermal management and mechanical thromboprophylaxis are also essential components of perioperative VTE prevention. Maintaining normothermia, warming intravenous fluids, and applying intermittent pneumatic compression devices may help reduce venous stasis and coagulation activation.^[31,32]^

In the present case, the patient underwent P-PECD under general anesthesia. Although P-PECD is a minimally invasive spinal procedure and no major intraoperative complications were observed, several perioperative factors may have increased her susceptibility to thromboembolic events. The patient was 65 years old and had multiple comorbidities, including hypertension, hyperlipidemia, coronary atherosclerotic heart disease, and a history of coronary stent implantation. In addition, perioperative adjustment of her long-term antiplatelet therapy and early postoperative mobility restriction may have further influenced her thrombotic risk. Therefore, individualized VTE risk assessment and appropriate mechanical prophylaxis should be considered for elderly patients with multiple comorbidities undergoing minimally invasive spinal procedures.

General anesthesia is commonly used during endoscopic cervical discectomy. Previous studies have suggested that general anesthesia may be associated with an increased incidence of postoperative thrombotic events compared with regional anesthesia in certain surgical populations.^[33,34]^ However, the independent contribution of anesthesia type to postoperative APE remains uncertain. In this patient, general anesthesia was selected according to the requirements of P-PECD, and it should be considered one of the perioperative factors within the overall risk profile rather than a direct cause of APE.

### 4.4. Postoperative standardization of anticoagulation and anti-infection management

In patients at high risk of pulmonary embolism, prompt anticoagulation is essential to ensure safety. The Caprini risk assessment model is widely used for perioperative VTE risk stratification. According to the risk categories shown in Table 3, a score of 0 indicates very low risk, scores of 1 to 2 indicate low risk, scores of 3 to 4 indicate intermediate risk, scores of 5 to 8 indicate high risk, and scores >8 indicate extremely high risk. Patients at increased risk should receive appropriate thromboprophylaxis according to their individual bleeding and thrombotic risks.^[35]^ In this case, the patient had a Caprini score of 8 both on admission and on the 1st postoperative day, corresponding to the upper limit of the high-risk category (5–8 points). Arterial ultrasound of both lower limbs revealed multiple atherosclerotic plaques. Therefore, the patient received postoperative intermittent pneumatic compression and pharmacological thromboprophylaxis to prevent venous thrombosis.

**Table 3:** The 2005 Caprini risk assessment models for deep vein thrombosis.

Each risk factor represents 1 point		Each risk factor represents 2 points	Each risk factor represents 3 points	Each risk factor represents 5 points
Age 41–60 yr		Age 61–74 yr	Age over 74 yr	Elective major lower extremity arthroplasty
Minor surgery planned		Arthroscopic surgery	History of DVT/PE	Hip, pelvis, or leg fracture (<1 mo)
History of prior major surgery (<1 mo)		Malignancy (present or previous)	Family history of thrombosis	Stroke(<1 mo)
Varicose veins		Major surgery (>45 min)	Positive factor V Leiden	Multiple trauma (<1 mo)
History of inflammatory bowel disease		Laparoscopic surgery (>45 min)	Positive prothrombin 20210A	Acute spinal cord injury (paralysis) (<1 mo)
Swollen legs (current)		Patient confined to bed (>72 h)	Elevated serum homocysteine	
Obesity (BMI >25)		Immobilizing plaster cast (<1 mo)	Positive lupus anticoagulant	
Acute myocardial infarction		Central venous access	Elevated anticardiolipin antibodies	
Congestive heart failure (<1 mo)			Heparin-induced thrombocytopenia	
Sepsis (<1 month)			Other congenital or acquired thrombophilia	
Serious lung disease, incl. pneumonia (<1 month)				
Abnormal pulmonary function (COPD)				
Medical patient currently at bed rest				
For women only	Oral contraceptives or hormone replacement therapy			
	Pregnancy or postpartum (<1 mo)			
	History of unexplained stillborn infant, recurrent spontaneous abortion (≥3), premature birth with toxemia, or growth-restricted infant			

Very low risk: 0 points; low risk: 1 to 2 points; intermediate risk: 3 to 4 points; high risk: 5 to 8 points; extremely high risk: >8 points.

BMI = body mass index, COPD = chronic obstructive pulmonary disease, DVT = deep venous thrombosis, PE = pulmonary embolism.

The inflammatory response is a natural reaction to infection or injury, but it can also promote thrombosis, potentially leading to serious complications such as pulmonary embolism. Studies indicate that inflammatory markers like C-reactive protein are significantly elevated in patients with APE, underscoring the role of inflammation in its pathophysiology.^[36]^ Moreover, upregulation of the noncoding microRNA let-7b-5p in APE patients triggers endoplasmic reticulum stress by increasing stress-associated endoplasmic reticulum protein 1 expression, which exacerbates pulmonary embolism and promotes inflammatory responses and apoptosis in lung epithelial cells.^[37]^ Although the patient’s body temperature was normal (36.3°C) postoperatively and no routine blood test was initially performed, a white blood cell count of 11.41 × 10^9^/L on postoperative day 4 indicated a possible infection. Postoperative infection may increase the risk of APE. Therefore, for patients undergoing endoscopic cervical discectomy, regular postoperative blood monitoring and proactive anti-infective treatment are recommended to promptly identify and manage infections, thereby reducing the incidence of inflammation-induced APE.

### 4.5. Timely and accurate diagnosis

The clinical presentation of APE is highly heterogeneous and varies with age, comorbidities, as well as the size, number, and location of emboli. APE may be completely asymptomatic or present with nonspecific symptoms such as chest pain, cough, syncope, palpitations, dyspnea, or hemoptysis. These manifestations can easily mimic other cardiopulmonary disorders, complicating early diagnosis.^[18,38]^ In this case, initial vital signs, including SpO_2_, blood glucose, and heart rate, were within normal limits when the patient 1st reported dizziness and chest tightness. Blood pressure was 106/72 mm Hg (compared to 124/75 mm Hg on admission), dropped to 92/54 mm Hg after 5 minutes, and later returned to 115/67 mm Hg. APE was not suspected at initial assessment. Subsequently, high-sensitivity cardiac troponin I, N-terminal pro-B-type natriuretic peptide, arterial blood gas analysis, and plasma D-dimer were promptly evaluated. Anticoagulation was initiated following abnormal findings, and the diagnosis was confirmed via venous Doppler ultrasound of both lower limbs, bedside echocardiography, and CTPA. The patient was transferred to the intensive care unit for further management to prevent clinical deterioration.

D-dimer, a marker of hypercoagulable and secondary fibrinolysis, is widely used to exclude thromboembolism, especially in diagnosing APE and assessing thrombolytic efficacy. It has a sensitivity exceeding 95% for ruling out APE.^[39,40]^ However, its specificity decreases with age. Using an age-adjusted cutoff (age × 0.01 mg/L for patients >50 years) can improve specificity to 62%, 50%, 44%, and 35% for age groups 51 to 60, 61 to 70, 71 to 80, and >80 years, respectively, while maintaining 97% sensitivity.^[41]^ Notably, syncope, which was the initial symptom in this patient, may be the only or 1st manifestation of APE. Hypotension is also an important clinical sign.^[18,42]^ Therefore, accurate diagnosis of APE requires integration of symptoms, physical signs, laboratory tests, and imaging. Clinical probability scores such as the Wells score, Geneva score, or the “YEARS” algorithm can be used for risk stratification. In patients with suspected APE, lower-extremity venous Doppler ultrasound and echocardiography may provide additional diagnostic information, whereas CTPA should be performed promptly when indicated to confirm the diagnosis.

### 4.6. Early and effective treatment

For patients with a confirmed diagnosis, general supportive therapy and close monitoring of the respiratory and circulatory status are essential. Parameters including respiration, heart rate, blood pressure, oxygen saturation, electrocardiogram, and arterial blood gas should be closely observed. Active respiratory and circulatory support is required, including appropriate fluid resuscitation, and vasoactive medications such as norepinephrine and dobutamine are needed. Regarding anticoagulation, parenteral anticoagulation is recommended for patients with highly suspected APE. DOACs such as rivaroxaban and edoxaban are preferred over traditional vitamin K antagonists for the treatment of APE. Anticoagulation should be continued for at least 3 months. Long-term anticoagulation may be necessary in patients with genetic thrombophilia, persistent risk factors, recurrent VTEs, or active malignancy.^[40]^

In this case, the patient received effective supportive measures postoperatively, including level I monitoring, continuous cardiac observation, oxygen therapy, and anticoagulation therapy. Anticoagulation with low-molecular-weight heparin was initiated immediately upon suspicion of APE, alongside repeat measurements of D-dimer and other relevant indicators. After confirmation by CTPA, the patient was transferred to the intensive care unit for further management, where endotracheal intubation was prepared if needed for respiratory support. During the intensive care unit stay, treatments included anticoagulation, gastrointestinal protection, oxygen therapy, and nutritional support. The patient’s respiratory and circulatory conditions stabilized, with resolution of chest tightness and dizziness, allowing transfer back to the pain department ward. As the patient improved, low-molecular-weight heparin was discontinued, and her previous antiplatelet therapy was resumed. Discharge instructions included guidance regarding continuation of her prescribed medication regimen and follow-up in case of recurrent symptoms or discomfort.

## 5. Conclusion

This report describes a rare case of APE occurring after P-PECD in an elderly patient with multiple comorbidities. This case highlights that the risk of perioperative VTE cannot be completely eliminated even in minimally invasive spinal procedures. Several factors, including advanced age, hypertension, coronary atherosclerotic heart disease, perioperative modification of antithrombotic therapy, surgical stress, and postoperative activity restriction, may have contributed to the development of APE in this patient.

This case emphasizes the importance of systematic VTE prevention strategies in high-risk patients undergoing minimally invasive spinal surgery. Preoperatively, comprehensive thrombotic risk assessment and individualized prevention strategies should be considered. Intraoperatively, meticulous surgical techniques, appropriate operative management, and mechanical prophylaxis may help reduce thrombotic risk. Postoperatively, early mobilization, appropriate thromboprophylaxis, and close monitoring for unexplained symptoms such as decreased oxygen saturation, chest pain, dyspnea, or dizziness are essential. Prompt evaluation using laboratory tests and imaging examinations, including D-dimer assessment and CTPA when indicated, may facilitate early diagnosis and timely intervention.

Despite the advantages of minimally invasive spinal procedures, perioperative complications remain clinically important. This case provides practical insights into optimizing perioperative management of patients undergoing endoscopic cervical discectomy and suggests that individualized VTE risk assessment, prevention, monitoring, and treatment strategies may improve patient safety. However, the conclusions of this report are limited by the nature of a single case and the specific characteristics of this patient. Future prospective studies with larger sample sizes are required to determine the incidence, risk factors, and optimal prevention strategies for APE following minimally invasive spinal surgery.

## Acknowledgments

We sincerely thank all the medical staff in the department for their guidance and assistance during the management of this case, and thank the patient for her active cooperation and trust.

## Author contributions

**Conceptualization:** Wen Zhang, Xuebi Tian.

**Formal analysis:** Wen Zhang.

**Investigation:** Shangchen Yu, Zheng Li, Shuguang Yang, Chao Chen.

**Methodology:** Shangchen Yu.

**Project administration:** Wen Zhang, Xuebi Tian.

**Resources:** Shangchen Yu, Zheng Li, Shuguang Yang, Chao Chen, Kun Chen, Man Shi, Haiting Wang, Wen Zhang, Xuebi Tian.

**Software:** Siqi Zhou.

**Supervision:** Wen Zhang, Xuebi Tian.

**Writing – original draft:** Shangchen Yu, Siqi Zhou, Wen Zhang.

**Writing – review & editing:** Shangchen Yu, Wen Zhang, Xuebi Tian.
